# [Retracted] miR-338-3p regulates the proliferation, apoptosis and migration of SW480 cells by targeting MACC1

**DOI:** 10.3892/etm.2025.12880

**Published:** 2025-05-08

**Authors:** Mingliang Lu, Hua Huang, Jinhui Yang, Jun Li, Gongfang Zhao, Weihua Li, Xinhua Li, Guobin Liu, Li Wei, Baoping Shi, Chunping Zhao, Yan Fu

Exp Ther Med 17:2807–2814, 2019; DOI: 10.3892/etm.2019.7260

Following the publication of the above paper, it was drawn to the Editor’s attention by a concerned reader that, regarding the flow cytometric plots shown in Fig. 3 on p. 2811, the ‘Control’ and ‘miR-338-30 inhibitor’ data panels appeared to show similar groupings of dots across the four quadrants, which would not have been anticipated if these experiments had been performed discretely under different experimental conditions, suggesting a fundamental flaw either in the way in which these experiments were performed, or in how the results were outputted.

The Editor of *Experimental and Therapeutic Medicine* has decided that this paper should be retracted from the Journal on account of a lack of confidence in the presented data. The authors were asked for an explanation to account for these concerns, but the Editorial Office did not receive a reply. The Editor apologizes to the readership for any inconvenience caused.

